# An Intronic Variant in *CDKN1C* Gene Causing IMAGe Syndrome in an Iranian Girl

**DOI:** 10.1002/mgg3.70154

**Published:** 2025-11-11

**Authors:** Setila Dalili, Seyyedeh Azade Hoseini Nouri, Ameneh Sharifi, Reza Bayat, Saeid Talebi, Shahram Savad, Nazanin Medghalchi, Bahareh Rabbani, Nejat Mahdieh

**Affiliations:** ^1^ Pediatric Diseases Research Center Guilan University of Medical Sciences Rasht Iran; ^2^ Growth and Development Research Tehran University of Medical Sciences Tehran Iran; ^3^ Bahareh Medical Genetic Laboratory Tehran Iran; ^4^ Department of Medical Genetics, School of Medicine Iran University of Medical Sciences Tehran Iran; ^5^ Department of Medical Genetics Tehran University of Medical Sciences Tehran Iran; ^6^ Fraser International College (FIC) SFU Pathway Burnaby, British Columbia Canada; ^7^ Cardiogenetic Research Center Rajaie Cardiovascular Institute Tehran Iran; ^8^ Fetal and Pediatric Cardiovascular Research Center, Children's Medical Center Tehran University of Medical Sciences Tehran Iran

**Keywords:** *CDKN1C* gene, genetic diagnosis, IMAGe syndrome, paternal imprinting

## Abstract

**Introduction:**

IMAGe syndrome, a rare disorder caused by maternally inherited *CDKN1C* pathogenic variants, is characterized by intrauterine growth retardation (IUGR), metaphyseal dysplasia, adrenal hypoplasia congenita, and genitourinary abnormalities. We report a novel intronic *CDKN1C* variant in a 5‐year‐old Iranian girl with IMAGe syndrome.

**Materials and Methods:**

Clinical evaluations showed severe IUGR (birth weight 1850 g), disproportionate short stature (height 88 cm, −4.4 *Z* score), metaphyseal dysplasia, adrenal insufficiency (ACTH 1110 pg/mL, low cortisol), and dysmorphic features (frontal bossing, low‐set ears). Whole‐exome sequencing (WES) was performed to identify causative genetic variants.

**Results:**

WES revealed a heterozygous *CDKN1C* intronic variant, c.787+4A>T, absent from gnomAD, ExAC, and ClinVar. SpliceAI (score: 0.82) predicted disrupted splicing, potentially leading to a gain‐of‐function effect. The variant was consistently classified as a Variant of Uncertain Significance (VUS) according to ACMG/AMP 2015 guidelines with 2020 updates. No other pathogenic variants were identified in genes related to skeletal dysplasia, adrenal insufficiency, or growth retardation. Sanger sequencing confirmed maternal inheritance in the proband, her healthy mother, and grandfather, consistent with *CDKN1C* paternal imprinting.

**Discussion:**

This case broadens the genetic spectrum of IMAGe syndrome by identifying the first reported intronic *CDKN1C* variant associated with this condition. WES is crucial for diagnosis, and RNA analysis is needed to confirm the variant's functional impact. Rapid diagnosis is essential for managing life‐threatening adrenal insufficiency.

## Graphical abstract

A novel intronic CDKN1C variant (c.787+4A>T) was identified in an Iranian girl with IMAGe syndrome, expanding its genetic spectrum. This finding highlights the importance of whole‐exome sequencing for diagnosing rare disorders and managing life‐threatening adrenal insufficiency.

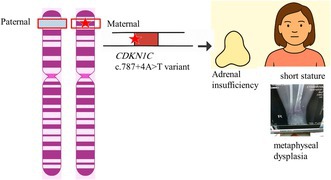

**Abbreviations**
ACMG: American College of Medical Genetics and Genomics
BWS: Beckwith‐Wiedemann syndrome
CADD: combined annotation dependent depletion
CNV: Copy number variant
EEG: electroencephalogram
FGR: fetal growth restriction
GH: growth hormone
IMAGe: intrauterine growth retardation, metaphyseal dysplasia, adrenal hypoplasia congenita, and genital anomalies
IUGR: intrauterine growth retardation
MAF: minor allele frequency
SNV: single‐nucleotide variants
VUS: variant of uncertain significance
WES: whole‐exome sequencing

## 1 Introduction

Intrauterine growth retardation, metaphyseal dysplasia, adrenal hypoplasia congenita, and genital anomalies (IMAGe syndrome) (OMIM #614732), a rare multisystem disorder, is characterized by intrauterine growth retardation (IUGR), metaphyseal dysplasia, adrenal hypoplasia congenita, and genitourinary abnormalities. Its heterogeneous clinical presentation, including skeletal anomalies (e.g., epiphyseal dysplasia, short stature, mesomelia, scoliosis), adrenal insufficiency, and dysmorphic features (e.g., frontal bossing, low‐set ears), poses significant diagnostic and management challenges. To date, only 31 cases from 19 families have been reported globally, highlighting its rarity (Logan et al. 2018; Çamtosun et al. 2021).

Among its defining features, metaphyseal dysplasia stands out prominently, often accompanied by additional skeletal abnormalities such as epiphyseal dysplasia, delayed bone age, short stature, mesomelia, short metacarpals/metatarsals, hallux valgus, scoliosis, hip dysplasia, osteopenia, and striated or gracile diaphysis (Yoo 2021; Pedreira et al. 2004; Bolomiti et al. 2021). Congenital adrenal hypoplasia, another hallmark of IMAGe syndrome, typically manifests within the first days of life. Meanwhile, urogenital anomalies, predominantly observed in males, may include cryptorchidism, micropenis, hypospadias, and chordee, with females often presenting with milder or absent abnormalities in this domain (Yoo 2021; Pedreira et al. 2004; Bolomiti et al. 2021). Furthermore, neurodevelopmental impairments such as hypotonia, developmental delays, and varying degrees of cognitive deficits have been reported in some affected individuals (Pedreira et al. 2004). Additional features encompassing hypercalciuria/hypercalcemia, craniosynostosis, cleft palate, scoliosis (Balasubramanian et al. 2010), and facial dysmorphism—including frontal bossing, low‐set ears, macrocephaly, micrognathia, high‐arched palate, short nose, and wide nasal bridge—further contribute to the clinical heterogeneity of IMAGe syndrome (Stokes et al. 2015).

The pathogenesis of IMAGe syndrome is primarily linked to heterozygous pathogenic variants in the *CDKN1C* gene inherited in an autosomal dominant manner exclusively through maternal transmission (Çamtosun et al. 2021; Stokes et al. 2015). Notably, specific missense pathogenic variants within the PCNA‐binding domain of *CDKN1C* disrupt normal protein function, culminating in aberrant growth inhibition and differentiation processes (Arboleda et al. 2012; Hamajima et al. 2013; Binder et al. 2020). In this study, we present the genetic and clinical profile of a 5‐year‐old Iranian girl exhibiting disproportionate short stature, multiple skeletal anomalies, dysmorphic features, and congenital adrenal insufficiency, indicative of IMAGe syndrome. Remarkably, her mother has experienced prior pregnancies marked by miscarriage and stillbirth, characterized by similar fetal presentations.

## 2 Materials and Methods

### 2.1 Clinical Assessments

A 5‐year‐old Iranian girl was referred to the Endocrinology Department at Rasht 17 Shahrivar Hospital, Iran, for evaluation of short stature. Clinical assessments included perinatal history review, physical examination, neurological evaluation, and laboratory tests to assess growth, adrenal function, and other biological markers. Imaging studies comprised abdominopelvic sonography to evaluate abdominal and pelvic organs, echocardiography to assess cardiac function, brain sonography, and CT scan to examine cranial structures. These investigations aimed to identify abnormalities consistent with IMAGe syndrome and rule out other conditions.

### 2.2 Genetic Analysis

#### 2.2.1 DNA Extraction and Whole‐Exome Sequencing (WES)

Genomic DNA was extracted from the proband's peripheral blood using standard protocols. Whole‐exome sequencing (WES) was performed to identify causal variants. Target enrichment was achieved using the Agilent SureSelect Human All Exon V7 kit, capturing coding exons and flanking intronic regions. Sequencing was conducted on an Illumina NovaSeq 6000 platform, generating paired‐end 150 bp reads with an average coverage depth of 100× for > 98% of targeted bases. Reads were aligned to the human reference genome (GRCh37/hg19) using BWA‐MEM (v0.7.17). Variant calling was performed with GATK HaplotypeCaller (v4.2), focusing on single‐nucleotide variants (SNVs) and small insertions/deletions (< 5 bp), with an analytical sensitivity of > 97%.

Variants were filtered based on a minor allele frequency (MAF) < 0.01 in population databases (gnomAD v2.1.1, ExAC, ESP6500). Pathogenicity was assessed using bioinformatics tools, including MutationTaster, SIFT, PROVEAN, FATHMM, and CADD (Combined Annotation Dependent Depletion). For intronic variants, splice prediction tools SpliceAI and dbscSNV evaluated potential splicing disruptions. Variants were classified per American College of Medical Genetics and Genomics (ACMG) guidelines. The identified *CDKN1C* variant was referenced to transcript NM_001122630.2, with potential nomenclature discrepancies. Karyotype analysis was performed to assess chromosomal composition and rule out structural abnormalities.

#### 2.2.2 Validation and Segregation Analysis

Sanger sequencing was used to confirm the identified *CDKN1C* variant in the proband, her mother, and her maternal grandfather. DNA from the father, aunt, and miscarried/stillborn fetuses was unavailable, limiting further family testing. Copy number variant (CNV) analysis was not included in the WES pipeline, and RNA analysis was not performed due to resource constraints.

## 3 Results

### 3.1 Clinical Features and Findings

**FIGURE 1:**
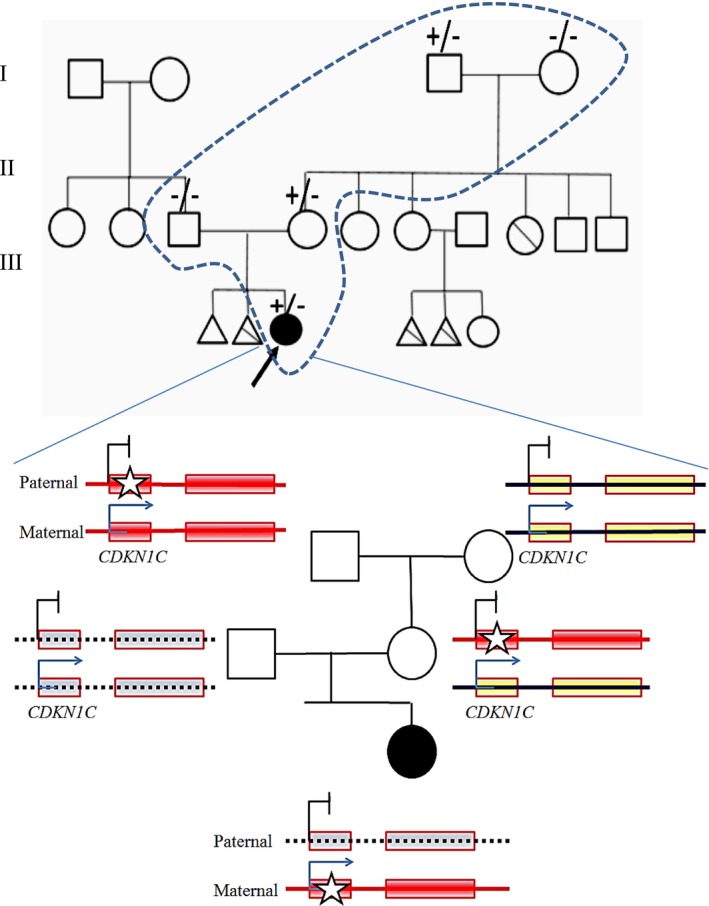
Pedigree showing the patient (5‐year‐old Iranian female), her parents, maternal grandfather, maternal aunt, and the mother's two prior pregnancies (intrauterine fetal demise at 38 weeks, termination at 20 weeks due to fetal anomalies). The proband, her mother, and maternal grandfather carry the heterozygous *CDKN1C* c.787+4A>T variant, confirmed by Sanger sequencing. The aunt's recurrent miscarriages suggest a possible carrier status, untested due to unavailable DNA. Imprinting at the *CDKN1C* locus involves methylation patterns, where the locus is unmethylated on the maternal allele and methylated on the paternal allele. Consequently, *CDKN1C* is paternally imprinted and maternally expressed. IMAGe syndrome results from a heterozygous variant inherited maternally. Therefore, if the maternal allele carries the mutation, the child will be affected, while a mutation in the paternal allele (which is imprinted) does not lead to an affected child. The shaded boxes indicate genes, and an asterisk (*) indicates the variant.

A 5‐year‐old Iranian girl, the only surviving child of non‐consanguineous parents (mother aged 25, healthy; father's health status unavailable), was referred to the Endocrinology Department at Rasht 17 Shahrivar Hospital, Iran, for short stature. The mother reported two prior pregnancies ending in fetal loss: the first at 38 weeks due to intrauterine fetal demise, with anomalies including polyhydramnios, low‐set ears, short limbs, and frontal bossing; the second at 18 weeks, terminated after ultrasound revealed a 46 XY karyotype, ventriculomegaly, low‐set ears, abnormal limb proportions, leg bowing, severe dwarfism, and skeletal dysplasia. The patient's maternal aunt also experienced recurrent miscarriages with similar fetal anomalies, suggesting a hereditary predisposition, though not diagnostic for IMAGe syndrome (Figure 1).

Perinatal assessment of the proband showed severe asymmetric IUGR, short limbs, mild polyhydramnios, and bilateral small choroidal plexus cysts on ultrasound. Delivered by cesarean section at 38 weeks, she had Apgar scores of 7 (1 min) and 9 (5 min), with a birth weight of 1850 g, a length of 43 cm, and a head circumference of 36.5 cm. Mild hypotonia, low‐set ears, and profound skin hyperpigmentation were noted. On day 3, she developed poor feeding, vomiting, dehydration, hypotension, acidosis, hypoglycemia (38 mg/dL), hyponatremia (127 mmol/L), and hyperkalemia (6.2 mmol/L). Adrenal ultrasound, elevated ACTH (1110 pg/mL), and low cortisol (0.6 μg/L) ruled out congenital adrenal hyperplasia, prompting fludrocortisone and hydrocortisone treatment, which resolved hyperpigmentation. Genital examination showed normal female anatomy without clitoromegaly.

**FIGURE 2:**
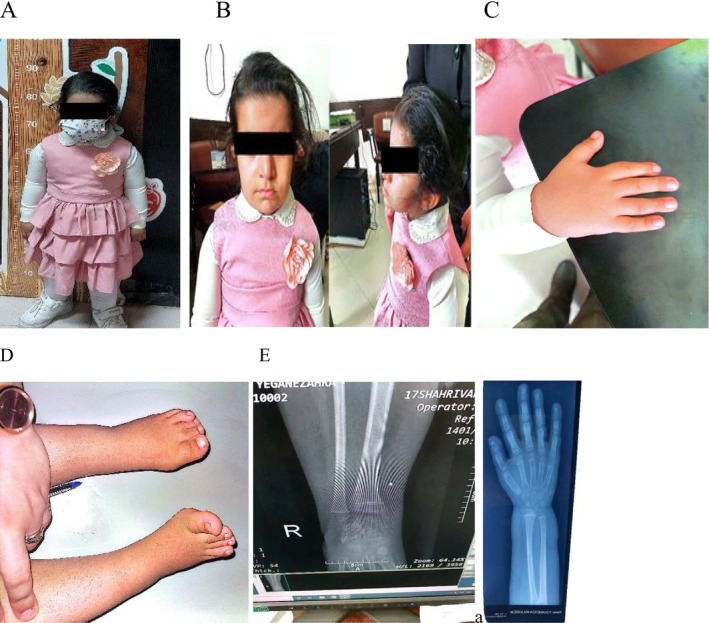
(A) Full‐body photograph at age 5, demonstrating disproportionate short stature (height: 88 cm, *Z* score −4.4; upper‐to‐lower body segment ratio: 1.8). (B) Clinical image highlighting facial dysmorphism, including frontal bossing, mild hypertropia of the right eye, low‐set ears, and a short neck, consistent with IMAGe syndrome. (C) Photograph of the right hand showing a shortened index finger. (D) Image of the feet demonstrating bilateral clinodactyly of the fourth toes, bending toward the third toes. (E) Radiographs of wrists and hips revealing osteonecrosis, metaphyseal dysplasia, and frayed bone edges.

At age 2, a hypoglycemic seizure led to Tegretol syrup treatment. Frequent infections caused multiple hospitalizations with vomiting, electrolyte imbalances, and adrenal crises, managed with stress‐dose corticosteroids. Initially delayed in developmental, motor, and speech skills, the patient achieved normal motor, language, and intelligence by age 5. Despite parental heights of 162 cm (mother) and 176 cm (father), her growth was severely compromised: at age 5, weight was 18.7 kg (49.6th percentile, *Z* score −0.01), height was 88 cm (0.1st percentile, *Z* score −4.4), and head circumference was 49 cm (25th percentile). She exhibited disproportionate short stature (upper‐to‐lower body segment ratio 1.8, higher than expected; Figure 2A), frontal bossing, mild hypertropia of the right eye, low‐set ears, a short neck (Figure 2B, full‐body image consistent with clinical description), short right index finger (Figure 2C), out‐toeing gait, bilateral clinodactyly of the fourth toes, and cognitive problem (Figure 2D). Dental carriers were noted. Neurological, chest, abdominal, spinal (no scoliosis), and genital examinations were normal, with breasts at Tanner stage 1 and no mucosal hyperpigmentation. Vital signs were normal.

Laboratory results showed elevated ACTH (1110 pg/mL), IGF‐1 (334.7 ng/mL), TSH (6.82 mIU/L), and potassium (5.8 mmol/L), with low 17‐hydroxyprogesterone (0.1 pg/mL), sodium (130 mmol/L), and cortisol (0.6 μg/L). Other parameters were normal, with no hypocalcemia or hypercalciuria. Bone age was consistent with 5.5 years. Radiographs revealed osteopenia, metaphyseal dysplasia, and frayed bone edges (Figure 2E). Abdominopelvic sonography showed bilateral adrenal enlargement (right‐sided prominence), consistent with adrenal hyperplasia, but normal renal and urinary tract findings. Echocardiography, brain sonography, CT scan, and audiometry were normal, with no hydrocephalus or hearing impairment. Karyotype analysis confirmed a normal female chromosomal pattern with a 46, XX karyotype, devoid of structural abnormalities or chromosomal rearrangements. The proband's clinical features were consistent with IMAGe syndrome.

### 3.2 Genetic Findings

WES identified a heterozygous intronic VUS variant in *CDKN1C* (NM_001122630.2: c.787+4A>T), absent from gnomAD, ExAC, 1000 Genomes, HGMD, and ClinVar. No other pathogenic variants were found in genes associated with skeletal dysplasias (e.g., *FGFR3*, *COL1A1*), adrenal insufficiency (e.g., *NR0B1*, *STAR*), or growth retardation (e.g., *IGF1R*). SpliceAI (score: 0.82, threshold > 0.5) and dbscSNV predicted disrupted splicing near the exon 3 splice acceptor site, potentially causing a gain‐of‐function effect. CADD (score: 23.4) and GERP++ supported pathogenicity, but the lack of functional studies classified the variant as a VUS per ACMG guidelines (PM2, PP3). Sanger sequencing confirmed the variant in the proband, her healthy mother, and maternal grandfather, consistent with maternal inheritance and *CDKN1C* paternal imprinting. The mother's and grandfather's healthy status aligns with the silenced paternal allele. DNA from the father, aunt, or miscarried fetuses was unavailable, limiting segregation analysis. Karyotype analysis confirmed a normal 46, XX karyotype without structural abnormalities, supporting the clinical diagnosis of IMAGe syndrome driven by the *CDKN1C* variant.

We conducted classification of intronic mutations in 7 BWS patients, all inherited from the mother, with splice variants and clinical findings detailed in Table 1. In a separate family comprising two affected children, sequence analysis of the patients revealed a C>G splice site mutation at nucleotide position 2611, presenting with similar clinical symptoms of exomphalos and ear lobe abnormalities as observed in other patients with *CDKN1C* variants associated with syndromes involving uncontrollable overgrowth. In the study conducted by Brioude et al., two families with a total of four patients, including three boys and one girl, were identified with the c.821‐9C>A and c.951+2T>C variants, respectively. All patients exhibited consistent symptoms such as macrosomia, macroglossia, and umbilical hernia.

**TABLE 1:** intronic variants of the *CDKN1C* gene and clinical features of the patients.

No. family	Patient	Ethnicity	Symptoms	Mutation (intron)	Disease	References
1	1	Spain	Mild macroglossia, flat vascular malformation in the glabella, Omphalocele, inguinal hernia	c.821‐2A>G (3)	BWS	Romanelli et al. (2010)
2	1	Japan	Overgrowth, Exomphalos, Macroglossia, Ear lobe abnormalities	c 821‐3C>G (3)	BWS	Lam et al. (1999)
	2	Japan	Overgrowth, Exomphalos, Macroglossia, earlobe abnormalities, hypoglycemia, and prune belly syndrome	c 821‐3C>G (3)	BWS	Lam et al. (1999)
3	1	Paris	Macrosomia, Macroglossia, Exomphalos, Visceromegaly, Neonatal Hypoglycemia	c.821‐9C>A (3)	BWS	Brioude et al. (2015)
	2	Paris	Macroglossia, umbilical hernia	c.821‐9C>A (3)	BWS	Brioude et al. (2015)
4	1	Paris	Macrosomia, Macroglossia, umbilical hernia, and ear anomalies	c.951+2T>C (4)	BWS	Brioude et al. (2015)
	2	Paris	Macrosomia, Macroglossia, umbilical hernia, and ear anomalies	c.951+2T>C (4)	BWS	Brioude et al. (2015)
5	1	Iran	Mild hypotonia, low‐set ears, skin hyperpigmentation, poor feeding, vomiting, dehydration, hypotension, acidosis, hypoglycemia, hyponatremia, and hyperkalemia	c.787+4A>T (2)	IMAGe syndrome	This study

Abbreviation: BWS, Beckwith‐Wiedemann syndrome.

### 3.3 Patient Management

The patient began hydrocortisone and fludrocortisone therapy at age 3 days, stabilizing sodium and potassium levels and resolving skin hyperpigmentation. Growth hormone (GH) therapy for detected GH deficiency showed no significant improvement in height. Hypothyroidism treatment was recently initiated, and Tegretol was continued under pediatric neurology supervision based on electroencephalogram (EEG) findings. Annual monitoring of height and weight, calcium and vitamin D supplementation, and referrals to orthopedic and ophthalmology specialists (for skeletal abnormalities and right eye hypertropia) are ongoing.

## 4 Discussion

IMAGe syndrome (OMIM #614732), characterized by IUGR, metaphyseal dysplasia, adrenal hypoplasia congenita, and genitourinary anomalies, is a rare disorder first described by Vilain et al. (1999). To date, 31 individuals from 19 families have been reported, with 18 having confirmed molecular diagnoses (Kim et al. 2018). This case report describes a 5‐year‐old Iranian girl with IMAGe syndrome associated with a novel intronic *CDKN1C* variant (c.787+4A>T), the first such variant linked to this condition, expanding its genetic spectrum. To the best of our knowledge, all reported intronic variants are associated with BWS. The present case adds to this growing body of literature and highlights the importance of recognizing and accurately diagnosing IMAGe syndrome in order to provide appropriate medical management and counseling for affected individuals and their families.

Intronic variants of a gene can potentially disrupt the normal splicing of the gene, leading to the production of an abnormal protein or no protein at all. They might also affect the stability or localization of the gene. In the same vein, intronic variants of a gene might also influence the interactions between the gene and other proteins or molecules. The patient's mother and aunt's history of miscarriages with similar fetal anomalies suggests she may carry the c.787+4A>T variant, supported by *CDKN1C*'s high placental expression and role in fetal growth restriction (Suntharalingham et al. 2019). Given the known imprinting status of *CDKN1C* (maternally expressed, paternally imprinted), segregation of the variant with disease within the family supports a pathogenic role. The variant was detected in the proband and her mother (carrier) but not expressed in the maternal grandfather, consistent with the expected parent‐of‐origin effect. This pattern fulfills the ACMG criterion PP1 supporting (segregation consistent with disease and gene mechanism). Thus, the revised ACMG criteria are PM2 + PP3 + PP1. This variant was identified in a family of northern Iranian origin and has not been reported in population databases (gnomAD v2.1.1, 1000 Genomes). Considering its absence in global datasets and our previous observation of other rare gene variants within ethnic subpopulations in our country, a founder effect cannot be excluded. However, current data are insufficient to confirm this hypothesis, and additional haplotype or population‐based studies are required to establish any founder relationship (Davoudi‐Dehaghani et al. 2013; Ramazani et al. 2008; Riazalhosseine et al. 2005; Cengiz et al. 2017).

It is noteworthy that both Beckwith‐Wiedemann syndrome (BWS) and IMAGe syndrome have been associated with mutations in the *CDKN1C* gene. Specifically, loss‐of‐function mutations in the *CDKN1C* gene have been implicated in the pathogenesis of 5%–10% of BWS patients (Eggermann et al. 2014), while gain‐of‐function mutations in this gene have been linked to IMAGe syndrome (Berland et al. 2022). The c.787+4A>T variant is predicted to affect splicing, which could theoretically result in a gain‐of‐function mechanism similar to that described in IMAGe‐associated missense variants within the PCNA‐binding domain. Unlike missense variants in the PCNA‐binding domain, which directly impair CDKN1C's interaction with PCNA and disrupt cell cycle regulation (Arboleda et al. 2012; Hamajima et al. 2013), this intronic variant may result in aberrant mRNA splicing, potentially producing a protein with enhanced inhibitory activity on cell cycle progression. The proband's phenotype, including severe IUGR, metaphyseal dysplasia, adrenal insufficiency, and dysmorphic features, aligns with previously reported IMAGe syndrome cases caused by PCNA‐binding domain variants. However, the absence of relative macrocephaly and hypercalciuria in this patient, which are variably reported in IMAGe syndrome (Kato et al. 2014), suggests potential phenotypic variability. This variability could stem from differences in how intronic variants affect CDKN1C expression or protein function compared to missense variants. For instance, altered splicing could lead to tissue‐specific expression changes, particularly in the placenta, where CDKN1C is highly expressed (Suntharalingham et al. 2019), potentially exacerbating IUGR. Although RNA validation is not yet available, the phenotype of the proband closely mirrors that of patients with confirmed gain‐of‐function *CDKN1C* variants, supporting the possibility of a similar functional mechanism through an alternative molecular route. Functional assays, including RT‐PCR and cDNA sequencing, are required to verify this hypothesis.

While gain‐of‐function missense variants in the PCNA‐binding domain are the primary known cause of IMAGe syndrome, this intronic variant suggests additional mechanisms. Disrupted splicing may lead to a truncated or modified CDKN1C protein with enhanced cyclin‐dependent kinase inhibition, amplifying growth restriction. Alternatively, the variant could affect non‐coding RNA interactions or regulatory elements within the intron, altering *CDKN1C* expression levels (Kornblihtt et al. 2013). Other mechanisms, such as epigenetic dysregulation or interactions with imprinting control regions, cannot be ruled out without functional studies. The maternal inheritance and paternal imprinting observed in this case align with CDKN1C's known expression pattern, supporting its role in IMAGe syndrome pathogenesis. However, the possibility of other genes or pathways contributing to IMAGe‐like phenotypes, as seen in POLE1‐related IMAGEI syndrome (Logan et al. 2018), warrants further investigation. Comparison with known *CDKN1C* intronic variants associated with BWS (Table 1) further highlights the unique nature of this variant. For example, c.821‐2A>G, c.821‐3C>G, and c.951+2T>C disrupt splicing near exons 3 and 4 and result in the overgrowth features characteristic of BWS, as opposed to the gain‐of‐function growth restriction seen in IMAGe syndrome.

Adrenal insufficiency, a hallmark of IMAGe syndrome, manifested neonatally in this patient, consistent with most cases, though childhood onset has been reported (Pedreira et al. 2004). Unlike a prior case with hearing loss (Balasubramanian et al. 2010), this patient had normal audiometry. Relative macrocephaly, noted in some IMAGe cases (Kim et al. 2018), was absent here in our patient. Growth hormone (GH) deficiency, observed in this patient, is variable in IMAGe syndrome, with poor response to GH therapy, as seen here and in other reports (Pedreira et al. 2004; Kato et al. 2014; Lienhardt et al. 2002). Given concerns about GH therapy's potential cancer risks (Bamba and Kanakatti Shankar 2022) and *CDKN1C*'s association with malignancies in BWS, caution is warranted, though no malignancy link is established for IMAGe syndrome beyond a single rhabdomyosarcoma case (Bolomiti et al. 2021). Hypothyroidism, recently detected in this patient, aligns with a prior report (Stokes et al. 2015), while the absence of hypercalciuria or nephrocalcinosis is consistent with other cases (Pedreira et al. 2004). Unlike IMAGEI syndrome, linked to *POLE1* pathogenic variants and immunodeficiency, this patient showed no immunological deficits (Logan et al. 2018). Prompt diagnosis of IMAGe syndrome enabled early intervention, critical for managing life‐threatening adrenal insufficiency. Since day 3, the patient received hydrocortisone and fludrocortisone, normalizing electrolyte imbalances and reducing hyperpigmentation. Despite GH therapy for growth deficiency, height gains were minimal, consistent with variable responses in IMAGe syndrome. Recent hypothyroidism treatment, ongoing seizure management with Tegretol, and supplementation with calcium and vitamin D support comprehensive care. Referrals to orthopedics for skeletal issues and ophthalmology for hypertropia ensure multidisciplinary management, with annual growth monitoring to track progress. Rapid WES helped to support the clinical diagnosis and to exclude other causes of adrenal hypoplasia, underscoring the utility of exome analysis in rare disorders.

The *CDKN1C* gene is expressed at higher levels in the placenta compared to adult tissues, which suggests that it may play a role in the development of fetal growth restriction (FGR) and supports the hypothesis that it is involved in the maintenance of pregnancy (Suntharalingham et al. 2019). The fact that the patient's mother had two previous pregnancies with embryos displaying similar anomalies, and the patient's aunt experienced repeated miscarriages, indicates that the mother and aunt are carriers of the mutated gene.

## 5 Conclusion

This case expands the genetic spectrum of IMAGe syndrome by reporting a novel intronic *CDKN1C* variant. The proband's phenotype underscores the importance of rapid diagnosis to manage life‐threatening adrenal insufficiency, particularly in females without genital ambiguity. Genetic testing targeting *CDKN1C* is critical for suspected IMAGe syndrome cases, and further studies are needed to confirm the functional impact of intronic variants.

## Author Contributions

**Setila Dalili:** formal analysis, conceptualization, supervision, writing – original draft, writing – review andediting, project administration. **Seyyedeh Azade Hoseini Nouri:** writing – review and editing, investigation, data curation. **Ameneh Sharifi:** formal analysis, methodology, writing – review and editing. **Reza Bayat:** investigation, methodology, writing – review and editing. **Saeid Talebi:** investigation, methodology, writing – review and editing. **Shahram Savad:** investigation, methodology, writing – review and editing. **Nazanin Medghalchi:** investigation, methodology, writing – review and editing. **Bahareh Rabbani:** investigation, methodology, writing – review and editing. **Nejat Mahdieh:** formal analysis, conceptualization, supervision, writing – original draft, writing – review and editing. All authors have read and agreed to the published version of the manuscript.

## Ethics Statement

The current study was conducted due to the Declaration of Helsinki and was approved by the Ethics Committee of Guilan University of Medical Sciences (IR.GUMS.REC.1402.184). Informed consent to participate in the study was obtained from the subject.

## Consent

Informed consent for publication was obtained from all the study participants.

## Conflicts of Interest

The authors declare no conflicts of interest.

## Data Availability

All data generated or analyzed during this study are included in this published paper.
